# 4-Nitro­phenyl 2-chloro­benzoate

**DOI:** 10.1107/S1600536812050362

**Published:** 2012-12-15

**Authors:** Asma Iqbal, Toheed Akhter, Humaira Masood Siddiqi, Zareen Akhter, Michael Bolte

**Affiliations:** a Department of Chemistry, Quaid-I-Azam University, Islamabad 45320, Pakistan; b Institut fur Anorganische Chemie, J. W. Goethe-Universität Frankfurt, Max-von-Laue-Strasse 7, 60438 Frankfurt/Main, Germany

## Abstract

The aromatic rings in the title compound, C_13_H_8_ClNO_4_, enclose a dihedral angle of 39.53 (3)°. The nitro group is almost coplanar with the ring to which it is attached [dihedral angle = 4.31 (1)°]. In the crystal, mol­ecules are connected by C—H⋯O hydrogen bonds into chains running along [001].

## Related literature

For the use of 4-nitro­phenyl-2-chloro­benzoate as a starting material for the synthesis of pain-relieving and anti-inflammatory drugs, see: Selvakumar *et al.* (2002 ▶); Jefford & Zaslona (1985 ▶). For a similar hydrogen-bonding pattern in a related structure, see: Akhter *et al.* (2012 ▶).
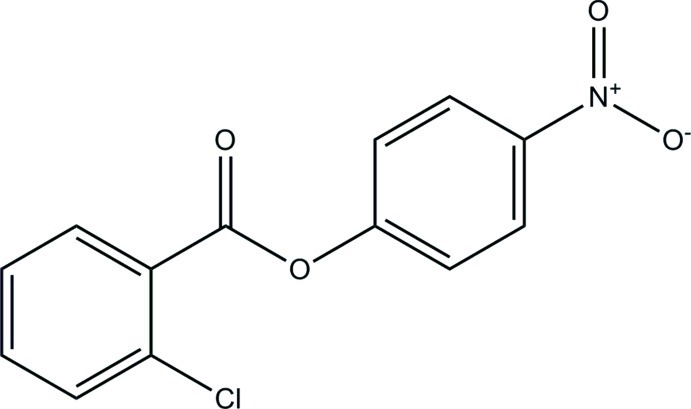


## Experimental

### 

#### Crystal data

C_13_H_8_ClNO_4_
*M* *_r_* = 277.65
Orthorhombic, 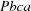

*a* = 11.4790 (4) Å
*b* = 14.0461 (5) Å
*c* = 14.3702 (7) Å
*V* = 2316.98 (16) Å^3^
*Z* = 8
Mo *K*α radiation
μ = 0.34 mm^−1^
*T* = 173 K
0.36 × 0.15 × 0.15 mm

#### Data collection

Stoe IPDS II two-circle diffractometer
Absorption correction: multi-scan (*X-AREA*; Stoe & Cie, 2001 ▶) *T*_min_ = 0.888, *T*_max_ = 0.951
54293 measured reflections
3251 independent reflections
3071 reflections with *I* > 2σ(*I*)
*R*_int_ = 0.057

#### Refinement

*R*[*F* ^2^ > 2σ(*F* ^2^)] = 0.044
*wR*(*F* ^2^) = 0.105
*S* = 1.14
3251 reflections
172 parameters
H-atom parameters constrained
Δρ_max_ = 0.32 e Å^−3^
Δρ_min_ = −0.41 e Å^−3^

### 

Data collection: *X-AREA* (Stoe & Cie, 2001 ▶); cell refinement: *X-AREA*; data reduction: *X-AREA*; program(s) used to solve structure: *SHELXS97* (Sheldrick, 2008 ▶); program(s) used to refine structure: *SHELXL97* (Sheldrick, 2008 ▶); molecular graphics: *XP* in *SHELXTL-Plus* (Sheldrick, 2008 ▶); software used to prepare material for publication: *SHELXL97*.

## Supplementary Material

Crystal structure: contains datablock(s) I, global. DOI: 10.1107/S1600536812050362/ff2092sup1.cif

Structure factors: contains datablock(s) I. DOI: 10.1107/S1600536812050362/ff2092Isup2.hkl

Supplementary material file. DOI: 10.1107/S1600536812050362/ff2092Isup3.cml

Additional supplementary materials:  crystallographic information; 3D view; checkCIF report

## Figures and Tables

**Table 1:** Hydrogen-bond geometry (Å, °)

*D*—H⋯*A*	*D*—H	H⋯*A*	*D*⋯*A*	*D*—H⋯*A*
C4—H4⋯O1^i^	0.95	2.48	3.3368 (19)	150

Symmetry code: (i) 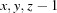.

**Fig. 1.**
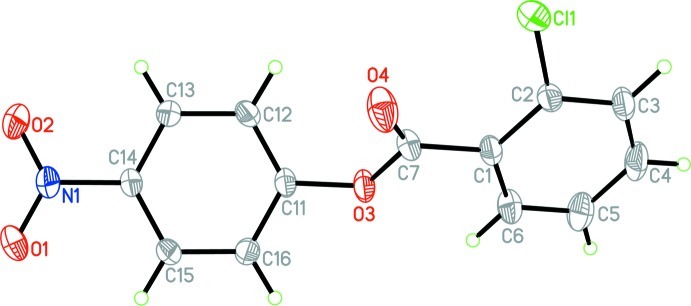
Perspective view of the title compound with the atom labeling scheme. Displacement ellipsoids are drawn at the 50% probability level.

## supplementary crystallographic information

### Comment

The aromatic esters, like 4-Nitrophenyl-2-chlorobenzoate, can be used as starting materials for the synthesis of several pain-relieving and anti-inflammatory drugs (Selvakumar *et al.*, 2002; Jefford & Zaslona, 1985).

The two aromatic rings in the title compound enclose a dihedral angle of 39.53 (3)°. The nitro group is almost coplanar with the phenyl ring to which it is attached [dihedral angle 4.31 (1)°]. In the crystal, the molecules are connected by C—H···O bonds to chains running along [0 0 1].

### Experimental

2-Chlorobenzoyl chloride was added drop wise to the solution of 4-nitrophenol (in a mixture of dry tetrahydrofuran (THF) and triethyl amine). The reaction mixture was stirred at room temperature for two hours and then was poured into the cold water. The oily product settled down after allowing the solution to stand for two hours, the supernatant liquid was decanted. The product was extracted from the solution by extraction with ethyl acetate, washed with 10% NaHCO_3_ solution to remove any traces of reactants and recrystallized from methanol. Yield 78%, m.p. 416–418 K.

### Refinement

All H atoms were initially located by difference Fourier synthesis. Subsequently all H atoms were refined using a riding model with C—H = 0.95 Å and with *U*_iso_(H) = 1.2*U*_eq_(C).

### Crystal data

**Table:**

C_13_H_8_ClNO_4_	*F*(000) = 1136
*M**_r_* = 277.65	*D*_x_ = 1.592 Mg m^−^^3^
Orthorhombic, *P**b**c**a*	Mo *K*α radiation, λ = 0.71073 Å
Hall symbol: -P 2ac 2ab	Cell parameters from 57790 reflections
*a* = 11.4790 (4) Å	θ = 3.2–30.1°
*b* = 14.0461 (5) Å	µ = 0.34 mm^−^^1^
*c* = 14.3702 (7) Å	*T* = 173 K
*V* = 2316.98 (16) Å^3^	Rod, colourless
*Z* = 8	0.36 × 0.15 × 0.15 mm

### Data collection

**Table:**

Stoe IPDS II two-circle diffractometer	3251 independent reflections
Radiation source: Genix 3D IµS microfocus X-ray source	3071 reflections with *I* > 2σ(*I*)
Genix 3D multilayer optics monochromator	*R*_int_ = 0.057
ω scans	θ_max_ = 29.7°, θ_min_ = 3.2°
Absorption correction: multi-scan (*X-AREA*; Stoe & Cie, 2001)	*h* = −15→15
*T*_min_ = 0.888, *T*_max_ = 0.951	*k* = −19→18
54293 measured reflections	*l* = −19→19

### Refinement

**Table:**

Refinement on *F*^2^	Primary atom site location: structure-invariant direct methods
Least-squares matrix: full	Secondary atom site location: difference Fourier map
*R*[*F*^2^ > 2σ(*F*^2^)] = 0.044	Hydrogen site location: inferred from neighbouring sites
*wR*(*F*^2^) = 0.105	H-atom parameters constrained
*S* = 1.14	*w* = 1/[σ^2^(*F*_o_^2^) + (0.0411*P*)^2^ + 1.2306*P*] where *P* = (*F*_o_^2^ + 2*F*_c_^2^)/3
3251 reflections	(Δ/σ)_max_ = 0.001
172 parameters	Δρ_max_ = 0.32 e Å^−^^3^
0 restraints	Δρ_min_ = −0.41 e Å^−^^3^

### Special details

**Table:**

Geometry. All e.s.d.'s (except the e.s.d. in the dihedral angle between two l.s. planes) are estimated using the full covariance matrix. The cell e.s.d.'s are taken into account individually in the estimation of e.s.d.'s in distances, angles and torsion angles; correlations between e.s.d.'s in cell parameters are only used when they are defined by crystal symmetry. An approximate (isotropic) treatment of cell e.s.d.'s is used for estimating e.s.d.'s involving l.s. planes.
Refinement. Refinement of *F*^2^ against ALL reflections. The weighted *R*-factor *wR* and goodness of fit *S* are based on *F*^2^, conventional *R*-factors *R* are based on *F*, with *F* set to zero for negative *F*^2^. The threshold expression of *F*^2^ > σ(*F*^2^) is used only for calculating *R*-factors(gt) *etc*. and is not relevant to the choice of reflections for refinement. *R*-factors based on *F*^2^ are statistically about twice as large as those based on *F*, and *R*- factors based on ALL data will be even larger.

### Fractional atomic coordinates and isotropic or equivalent isotropic displacement parameters (Å^2^)

**Table:**

	*x*	*y*	*z*	*U*_iso_*/*U*_eq_	
Cl1	0.66372 (3)	0.20873 (4)	0.41090 (3)	0.04536 (14)	
N1	0.85406 (11)	0.06742 (9)	1.00169 (8)	0.0282 (2)	
O1	0.93046 (12)	0.09364 (13)	1.05429 (8)	0.0557 (4)	
O2	0.76471 (11)	0.02876 (9)	1.02808 (8)	0.0417 (3)	
O3	0.92469 (8)	0.11781 (8)	0.62155 (6)	0.0280 (2)	
O4	0.76541 (11)	0.20800 (9)	0.59665 (7)	0.0403 (3)	
C1	0.88309 (12)	0.15657 (9)	0.46702 (8)	0.0236 (2)	
C2	0.80645 (13)	0.17488 (10)	0.39336 (9)	0.0272 (3)	
C3	0.84243 (15)	0.16241 (11)	0.30152 (10)	0.0352 (3)	
H3	0.7894	0.1738	0.2520	0.042*	
C4	0.95451 (17)	0.13369 (11)	0.28236 (10)	0.0372 (4)	
H4	0.9786	0.1251	0.2197	0.045*	
C5	1.03234 (16)	0.11733 (11)	0.35436 (10)	0.0357 (3)	
H5	1.1102	0.0989	0.3411	0.043*	
C6	0.99620 (14)	0.12797 (10)	0.44555 (9)	0.0297 (3)	
H6	1.0496	0.1155	0.4946	0.036*	
C7	0.84707 (12)	0.16577 (10)	0.56623 (9)	0.0246 (3)	
C11	0.90125 (11)	0.10896 (9)	0.71610 (8)	0.0219 (2)	
C12	0.79705 (12)	0.07089 (9)	0.74716 (9)	0.0242 (3)	
H12	0.7375	0.0541	0.7043	0.029*	
C13	0.78119 (11)	0.05777 (9)	0.84200 (9)	0.0232 (2)	
H13	0.7102	0.0325	0.8655	0.028*	
C14	0.87097 (11)	0.08221 (9)	0.90168 (8)	0.0214 (2)	
C15	0.97586 (11)	0.11932 (10)	0.87098 (9)	0.0233 (2)	
H15	1.0360	0.1350	0.9137	0.028*	
C16	0.99071 (11)	0.13298 (9)	0.77635 (9)	0.0235 (2)	
H16	1.0615	0.1586	0.7530	0.028*	

### Atomic displacement parameters (Å^2^)

**Table:**

	*U*^11^	*U*^22^	*U*^33^	*U*^12^	*U*^13^	*U*^23^
Cl1	0.02762 (19)	0.0729 (3)	0.0356 (2)	−0.00627 (18)	−0.00863 (14)	0.01702 (19)
N1	0.0323 (6)	0.0349 (6)	0.0174 (5)	−0.0006 (5)	0.0003 (4)	0.0019 (4)
O1	0.0494 (7)	0.0997 (12)	0.0180 (5)	−0.0213 (8)	−0.0083 (5)	0.0016 (6)
O2	0.0450 (7)	0.0552 (7)	0.0251 (5)	−0.0142 (6)	0.0084 (5)	0.0051 (5)
O3	0.0270 (5)	0.0425 (6)	0.0144 (4)	0.0076 (4)	0.0011 (3)	0.0034 (4)
O4	0.0442 (7)	0.0520 (7)	0.0246 (5)	0.0231 (5)	0.0002 (4)	−0.0003 (5)
C1	0.0312 (6)	0.0239 (6)	0.0155 (5)	−0.0014 (5)	−0.0013 (5)	0.0006 (4)
C2	0.0313 (7)	0.0293 (6)	0.0209 (6)	−0.0097 (5)	−0.0052 (5)	0.0051 (5)
C3	0.0509 (9)	0.0372 (8)	0.0174 (6)	−0.0186 (7)	−0.0075 (6)	0.0062 (5)
C4	0.0610 (10)	0.0341 (8)	0.0166 (6)	−0.0105 (7)	0.0064 (6)	−0.0017 (5)
C5	0.0484 (9)	0.0335 (7)	0.0251 (7)	0.0047 (7)	0.0102 (6)	−0.0009 (5)
C6	0.0362 (7)	0.0332 (7)	0.0196 (6)	0.0064 (6)	0.0015 (5)	−0.0004 (5)
C7	0.0293 (6)	0.0278 (6)	0.0167 (5)	0.0027 (5)	−0.0027 (5)	0.0002 (5)
C11	0.0243 (6)	0.0269 (6)	0.0146 (5)	0.0042 (5)	0.0005 (4)	0.0009 (4)
C12	0.0235 (6)	0.0304 (6)	0.0187 (5)	−0.0008 (5)	−0.0031 (4)	−0.0040 (5)
C13	0.0230 (6)	0.0263 (6)	0.0204 (5)	−0.0025 (5)	0.0011 (4)	−0.0008 (5)
C14	0.0249 (6)	0.0238 (6)	0.0155 (5)	0.0013 (5)	−0.0002 (4)	0.0005 (4)
C15	0.0214 (5)	0.0297 (6)	0.0189 (5)	−0.0001 (5)	−0.0034 (4)	0.0004 (5)
C16	0.0201 (5)	0.0298 (6)	0.0204 (5)	0.0009 (5)	0.0006 (4)	0.0031 (5)

### Geometric parameters (Å, º)

**Table:**

Cl1—C2	1.7245 (16)	C4—H4	0.9500
N1—O1	1.2149 (17)	C5—C6	1.3827 (19)
N1—O2	1.2210 (16)	C5—H5	0.9500
N1—C14	1.4650 (16)	C6—H6	0.9500
O3—C7	1.3710 (16)	C11—C12	1.3842 (18)
O3—C11	1.3906 (14)	C11—C16	1.3849 (18)
O4—C7	1.1923 (17)	C12—C13	1.3873 (18)
C1—C6	1.394 (2)	C12—H12	0.9500
C1—C2	1.4001 (18)	C13—C14	1.3839 (18)
C1—C7	1.4901 (17)	C13—H13	0.9500
C2—C3	1.394 (2)	C14—C15	1.3842 (18)
C3—C4	1.376 (3)	C15—C16	1.3839 (17)
C3—H3	0.9500	C15—H15	0.9500
C4—C5	1.386 (2)	C16—H16	0.9500
			
O1—N1—O2	123.23 (12)	O4—C7—O3	122.88 (12)
O1—N1—C14	118.15 (12)	O4—C7—C1	127.74 (12)
O2—N1—C14	118.63 (12)	O3—C7—C1	109.38 (11)
C7—O3—C11	118.99 (10)	C12—C11—C16	122.23 (11)
C6—C1—C2	118.10 (12)	C12—C11—O3	121.12 (11)
C6—C1—C7	119.68 (12)	C16—C11—O3	116.45 (11)
C2—C1—C7	122.21 (13)	C11—C12—C13	118.78 (12)
C3—C2—C1	120.43 (14)	C11—C12—H12	120.6
C3—C2—Cl1	117.03 (11)	C13—C12—H12	120.6
C1—C2—Cl1	122.49 (11)	C14—C13—C12	118.56 (12)
C4—C3—C2	120.22 (14)	C14—C13—H13	120.7
C4—C3—H3	119.9	C12—C13—H13	120.7
C2—C3—H3	119.9	C13—C14—C15	122.93 (11)
C3—C4—C5	120.12 (13)	C13—C14—N1	118.30 (12)
C3—C4—H4	119.9	C15—C14—N1	118.77 (11)
C5—C4—H4	119.9	C16—C15—C14	118.20 (11)
C6—C5—C4	119.75 (15)	C16—C15—H15	120.9
C6—C5—H5	120.1	C14—C15—H15	120.9
C4—C5—H5	120.1	C15—C16—C11	119.28 (12)
C5—C6—C1	121.36 (14)	C15—C16—H16	120.4
C5—C6—H6	119.3	C11—C16—H16	120.4
C1—C6—H6	119.3		
			
C6—C1—C2—C3	1.5 (2)	C7—O3—C11—C12	54.46 (18)
C7—C1—C2—C3	−177.88 (13)	C7—O3—C11—C16	−130.52 (13)
C6—C1—C2—Cl1	178.88 (11)	C16—C11—C12—C13	1.0 (2)
C7—C1—C2—Cl1	−0.49 (19)	O3—C11—C12—C13	175.74 (12)
C1—C2—C3—C4	−1.2 (2)	C11—C12—C13—C14	−0.81 (19)
Cl1—C2—C3—C4	−178.77 (12)	C12—C13—C14—C15	0.1 (2)
C2—C3—C4—C5	−0.2 (2)	C12—C13—C14—N1	−179.49 (12)
C3—C4—C5—C6	1.4 (2)	O1—N1—C14—C13	−176.12 (15)
C4—C5—C6—C1	−1.1 (2)	O2—N1—C14—C13	4.49 (19)
C2—C1—C6—C5	−0.3 (2)	O1—N1—C14—C15	4.3 (2)
C7—C1—C6—C5	179.08 (14)	O2—N1—C14—C15	−175.11 (13)
C11—O3—C7—O4	7.1 (2)	C13—C14—C15—C16	0.4 (2)
C11—O3—C7—C1	−173.56 (11)	N1—C14—C15—C16	−179.97 (12)
C6—C1—C7—O4	162.13 (16)	C14—C15—C16—C11	−0.3 (2)
C2—C1—C7—O4	−18.5 (2)	C12—C11—C16—C15	−0.5 (2)
C6—C1—C7—O3	−17.14 (18)	O3—C11—C16—C15	−175.43 (12)
C2—C1—C7—O3	162.22 (12)		

### Hydrogen-bond geometry (Å, º)

**Table:**

*D*—H···*A*	*D*—H	H···*A*	*D*···*A*	*D*—H···*A*
C4—H4···O1^i^	0.95	2.48	3.3368 (19)	150

Symmetry code: (i) *x*, *y*, *z*−1.
